# A Velocity-Level Bi-Criteria Optimization Scheme for Coordinated Path Tracking of Dual Robot Manipulators Using Recurrent Neural Network

**DOI:** 10.3389/fnbot.2017.00047

**Published:** 2017-09-04

**Authors:** Lin Xiao, Yongsheng Zhang, Bolin Liao, Zhijun Zhang, Lei Ding, Long Jin

**Affiliations:** ^1^College of Information Science and Engineering, Jishou University, Jishou, China; ^2^School of Automation Science and Engineering, South China University of Technology, Guangzhou, China; ^3^School of Information Science and Engineering, Lanzhou University, Lanzhou, China

**Keywords:** dual robot manipulators, bi-criteria optimization scheme, recurrent neural network, quadratic program, repetitive motion

## Abstract

A dual-robot system is a robotic device composed of two robot arms. To eliminate the joint-angle drift and prevent the occurrence of high joint velocity, a velocity-level bi-criteria optimization scheme, which includes two criteria (i.e., the minimum velocity norm and the repetitive motion), is proposed and investigated for coordinated path tracking of dual robot manipulators. Specifically, to realize the coordinated path tracking of dual robot manipulators, two subschemes are first presented for the left and right robot manipulators. After that, such two subschemes are reformulated as two general quadratic programs (QPs), which can be formulated as one unified QP. A recurrent neural network (RNN) is thus presented to solve effectively the unified QP problem. At last, computer simulation results based on a dual three-link planar manipulator further validate the feasibility and the efficacy of the velocity-level optimization scheme for coordinated path tracking using the recurrent neural network.

## 1 Introduction

Robot manipulators were widely investigated and applied to many fields (Jin et al., 2017; Zhang and Zhang, 2012; Xiao and Zhang, 2013, 2014a; Jin and Zhang, 2015; Zhang et al., 2015; Yamada et al., 2016), such as human–robot interaction, path tracking, industrial manufacturing, military, repetitive motion, and so on. Many researches have been focused on this topic, and various kinds of robot manipulators have been developed and investigated (Li et al., 2012, 2014, 2017; Xiao and Zhang, 2013; Jin and Zhang, 2015; Zhang et al., 2015). As far as we know, there are some manipulation tasks (including large, heavy, awkwardly sized payloads) that cannot be fulfilled by only a single robot manipulator. In contrast, dual robot manipulators can not only complete some common tasks but also can finish some complex and dangerous things that the single robot manipulator is usually hard to finish (Zhang and Li, 2017; Li et al., 2012, 2014; Jin and Zhang, 2015). In addition, dual robot manipulators have been successfully applied to various applications (Jin and Li, 2016; Zhang et al., 2013, 2015; Xiao and Zhang, 2014b; Jin and Zhang, 2015; Jin et al., 2016a), e.g., load transport, cooperative assembly, dextrous grasping, coordinate welding. Therefore, using dual robot manipulators to collectively conduct complicated tasks is becoming increasingly popular.

It is well known that inverse kinematics of robot manipulators (including dual manipulators) is a much more difficult problem than forward kinematics, but it is a fundamental issue in the field of robotics (also including dual robot manipulators). Generally speaking, there are two types of good methods for addressing the inverse kinematic problem. One is based on the pseudoinverse method that includes a homogeneous solution and a specific minimum-norm solution (Klein and Kee, 1989; Klein and Ahmed, 1995). However, the traditional pseudoinverse method needs to compute the inverse/pseudoinverse of matrices, which usually costs a lot of time. In addition, this method would lead to the joint angle drift when the end-effector completes a repetitive motion (Klein and Ahmed, 1995). The second method is based on optimization techniques, which treat performance criteria as objective functions (Jin and Li, 2016; Zhang et al., 2004; Guo and Zhang, 2012; Xiao and Zhang, 2013, 2014a, 2016). Among the existing schemes, single performance criterion is widely used to control the motion of manipulators at different joint levels, such as repetitive motion (Xiao and Zhang, 2013, 2014a), manipulability (Jin and Li, 2016), obstacle avoidance (Xiao and Zhang, 2016), minimum velocity norm (Guo and Zhang, 2012), and minimum torque norm (Zhang et al., 2004).

It is worth pointing out that single criterion optimization schemes cannot satisfy multiple requirements in practical applications, so dual-criteria optimization schemes are needed (Hou et al., 2010). Besides, considering the importance of the repetitive motion control for dual robot manipulators, it also requires an effective criterion for solving the joint-angle drift problem of dual robot manipulators in practical applications (Xiao and Zhang, 2013, 2014a; Zhang et al., 2013). To satisfy the above requirements, in this article, a novel bi-criteria optimization scheme is presented and investigated for coordinated path tracking of dual robot manipulators at the joint velocity level, of which the bi-criteria consist of the minimum velocity motion (MVN) and the repetitive motion (RM). Note that the proposed optimization scheme consists of two subschemes (corresponding to the left and right manipulators). Besides, such two subschemes can be rewritten as two general quadratic programs (QPs), which is further integrated into one QP formulation.

There are a lot of methods to solve the above QP problems, such as numerical algorithms, recurrent neural networks (RNN), and so on. Although the numerical algorithms can iterate good solutions, they are not suitable for real-time implementations due to their series characteristic and computational complexity. As an efficient computation tool, the neural network approach has several potential advantages in real-time applications (Li et al., 2013a,b; Li and Li, 2014; Xiao and Zhang, 2014c; Xiao, 2015, 2016a,b; Xiao and Lu, 2015; Jin et al., 2016b, 2017; Xiao and Liao, 2016), such as parallel processing, hardware implementation ability, and distributed storage. For example, a gradient-based neural network (GNN) has been widely used to solve various challenging mathematical problems (Zhang et al., 2009; Xiao and Zhang, 2011; Yi et al., 2011; Li et al., 2013c; Xiao, 2016c). Considering the advantages of this method, GNN is developed and applied for solving the proposed bi-criteria optimization scheme and the unified QP problem. Finally, on the basis of a dual three-link planar manipulator, we conduct circular path tracking simulations using such a GNN model and the proposed bi-criteria optimization scheme. The computer simulation results further verify the feasibility and effectiveness of the proposed scheme for coordinated path tracking of dual robot manipulators using the recurrent neural network.

## 2 Preliminaries

The forward kinematic equations of the robot manipulators at the position level and the velocity level can be expressed, respectively, as follows (Jin et al., 2017; Zhang and Zhang, 2012; Xiao and Zhang, 2013, 2014a; Jin and Zhang, 2015; Zhang et al., 2015):

(1) r(t)=f(θ(t)),

(2) $$ṙ\left(t\right)=J\left(\theta \right)\dot{\theta }\left(t\right),$$

where *θ*(*t*) ∈ *R^n^* and $\dot{\theta }\left(t\right)\in R^{n}$ denote the joint position vector and the joint velocity vector, respectively; *r*(*t*) ∈ *R^m^* and $ṙ\left(t\right)\in R^{m}$ denote the end-effector position vector and the end-effector velocity vector, respectively; Jacobian matrix $J\left(\theta \right)=\partial f\left(\theta \left(t\right)\right)∕\partial \theta \in R^{m\times n}$; and *f* (⋅) denotes a smooth non-linear function.

For example, for a three-link planar robot manipulator, we can readily get the forward-kinematic equation (the independent variable *t* is omitted for presentation convenience):

$$r=\left[\begin{array}{c} r_{\text{X}}\\ r_{\text{Y}} \end{array}\right]=\left[\begin{array}{c} l_{1}c_{1}+l_{2}c_{2}+l_{3}c_{3}\\ l_{1}s_{1}+l_{2}s_{2}+l_{3}s_{3} \end{array}\right]=f\left(\theta \right),$$

where *θ* = [*θ*_1_, *θ*_2_, *θ*_3_]^T^ ∈ *R*^3^, *r* ∈ *R*^2^, *l*_1_ denotes the length of the first link, *l*_2_ denotes the length of the second link, and *l*_3_ denotes the length of the third link. In addition, the variables depicted in the above are defined as

$$\begin{array}{ll} c_{1} & =\cos\left(\theta_{1}\right),\;s_{1}=\sin\left(\theta_{1}\right),\\ c_{2} & =\cos\left(\theta_{1}+\theta_{2}\right),\;s_{2}=\sin\left(\theta_{1}+\theta_{2}\right),\\ c_{3} & =\cos\left(\theta_{1}+\theta_{2}+\theta_{3}\right),\;s_{3}=\sin\left(\theta_{1}+\theta_{2}+\theta_{3}\right). \end{array}$$

The Jacobian matrix of *f* (⋅) can be solved in this situation by differentiating (1):

(3) $$J=\left[\begin{array}{ccc} -l_{1}s_{1}-l_{2}s_{2}-l_{3}s_{3} & -l_{2}s_{2}-l_{3}s_{3} & -l_{3}s_{3}\\ l_{1}c_{1}+l_{2}c_{2}+l_{3}c_{3} & l_{2}c_{2}+l_{3}c_{3} & l_{3}c_{3} \end{array}\right]\;.$$

Note that, in this article, we are concerned with the dual robot arms. Without loss of generality, one is called the left manipulator and the other is called the right manipulator for convenience. Therefore, the variables of the left and right robot manipulators of dual arms are correspondingly marked by subscripts _l_ and _r_. For example, variables *θ*_l_ and *θ*_r_ denote the joint position vectors of the left and right robot manipulators of dual arms, respectively. In Section 5, we set *l*_1_ = *l*_2_ = *l*_3_ = 1 m.

## 3 Scheme Formulation

For simplicity, the bi-criteria scheme of one robot manipulator is firstly proposed. To integrate the optimization criteria of the minimum velocity norm (MVN) and the repetitive motion (RM), a bi-criteria optimization objective at the velocity level is designed as

(4) $$\text{minimize}\;\;\;\;\;∥{\dot{\theta }}_{\text{l}∕\text{r}}{∥}_{2}^{2}∕2+∥{\dot{\theta }}_{\text{l}∕\text{r}}+q_{\text{l}∕\text{r}}{∥}_{2}^{2}∕2,$$

where $q_{\text{l}∕\text{r}}=\epsilon \left(\theta_{\text{l}∕\text{r}}-\theta_{\text{l}∕\text{r}}\left(0\right)\right)$ with ϵ > 0. Besides, performance index $∥{\dot{\theta }}_{\text{l}∕\text{r}}{∥}_{2}^{2}$ can achieve the minimum velocity motion of robot manipulators, and performance index $∥{\dot{\theta }}_{\text{l}∕\text{r}}+q_{\text{l}∕\text{r}}{∥}_{2}^{2}∕2$ can complete the repetitive motion task at the joint velocity level.

For the left robot manipulator, considering the forward kinematics equation and the above bi-criteria optimization objective, the bi-criteria optimization scheme can be formulated as below:

(5) $$\text{minimize}\;\;\;\;\;∥{\dot{\theta }}_{\text{l}}{∥}_{2}^{2}∕2+∥{\dot{\theta }}_{\text{l}}+q_{\text{l}}{∥}_{2}^{2}∕2,$$

(6) $$\text{subject to}\;\;\;\;J_{\text{l}}\left(\theta \right){\dot{\theta }}_{\text{l}}={ṙ}_{\text{l}},$$

where ${\dot{\theta }}_{\text{l}}$, *q*_l_, *J*_l_(*θ*), and ${ṙ}_{\text{l}}$ are defined the same as before, but belong to the variables of the left robot manipulator. Equation (5) uses the bi-criteria optimization objective (equation (4)); and equation (6) is the forward kinematics equation (2) of the left robot manipulator of dual arms.

For the right robot manipulator, the bi-criteria optimization scheme can be formulated as below in the same way:

(7) $$\text{minimize}\;\;\;\;\;∥{\dot{\theta }}_{\text{r}}{∥}_{2}^{2}∕2+∥{\dot{\theta }}_{\text{r}}+q_{\text{r}}{∥}_{2}^{2}∕2,$$

(8) $$\text{subject to}\;\;\;\;J_{\text{r}}\left(\theta \right){\dot{\theta }}_{\text{r}}={ṙ}_{\text{r}},$$

where ${\dot{\theta }}_{\text{r}}$, *q*_r_, *J*_r_(*θ*), and ${ṙ}_{\text{r}}$ are defined the same as before, but belong to the variables of the right robot manipulator.

## 4 QP Reformulation and Unification

In this section, to obtain two standard QP formulations, the proposed subschemes are rewritten as two QPs, which can be unified into one QP problem.

(1) Conversion of MVN criterion: according to definition of two norms, minimizing $∥{\dot{\theta }}_{\text{l}}{∥}_{2}^{2}∕2$ in the first term of equation (5) for the left robot manipulator is equivalent to
  (9) $$\text{minimize}\;\;\;\;\;\frac{{\dot{\theta }}_{\text{l}}^{\text{T}}I{\dot{\theta }}_{\text{l}}}{2},$$
  where *I* ∈ *R^n×n^* denotes an identity matrix.
  Similarly, MVN criterion $∥{\dot{\theta }}_{\text{r}}{∥}_{2}^{2}∕2$ in the first term of equation (7) for the right robot manipulator is equivalent to
  (10) $$\text{minimize}\;\;\;\;\;\frac{{\dot{\theta }}_{\text{r}}^{\text{T}}I{\dot{\theta }}_{\text{r}}}{2}.$$
(2) Conversion of RM criterion: the RM criterion $∥{\dot{\theta }}_{\text{l}}+q_{\text{l}}{∥}_{2}^{2}∕2$ in the second term of equation (5) for the left robot manipulator is rewritten equivalently as
  (11) $$\text{minimize}\;\;\;\;\;\frac{{\left({\dot{\theta }}_{\text{l}}+q_{\text{l}}\right)}^{\text{T}}\left({\dot{\theta }}_{\text{l}}+q_{\text{l}}\right)}{2},$$
  which is further equivalent to the following form:
  (12) $$\text{minimize}\;\;\;\;\;\frac{{\dot{\theta }}_{\text{l}}^{\text{T}}I{\dot{\theta }}_{\text{l}}+2q_{\text{l}}^{\text{T}}{\dot{\theta }}_{\text{l}}+q_{\text{l}}^{\text{T}}q_{\text{l}}}{2},$$
  where $q_{\text{l}}^{\text{T}}q_{\text{l}}$ can be deemed as a constant with respect to optimization variable $\dot{\theta }$ and can be ignored during minimization. Thus, the RM criterion $∥{\dot{\theta }}_{\text{l}}+q_{\text{l}}{∥}_{2}^{2}∕2$ of the left robot manipulator is finally equivalent to the following form:
  (13) $$\text{minimize}\;\;\;\;\;\frac{{\dot{\theta }}_{\text{l}}^{\text{T}}I{\dot{\theta }}_{\text{l}}+2q_{\text{l}}^{\text{T}}{\dot{\theta }}_{\text{l}}}{2}.$$

Similarly, the RM criterion $∥{\dot{\theta }}_{\text{r}}+q_{\text{r}}{∥}_{2}^{2}∕2$ of the right robot manipulator can be equivalent to the following form:

(14) $$\text{minimize}\;\;\;\;\;\frac{{\dot{\theta }}_{\text{r}}^{\text{T}}I{\dot{\theta }}_{\text{r}}+2q_{\text{r}}^{\text{T}}{\dot{\theta }}_{\text{r}}}{2}.$$

Thus, through the above conversion, the bi-criteria optimization subscheme for the left robot manipulator can be formulated as the following standard QP:

(15) $$\text{minimize}\;\;\;\;\;x_{\text{l}}^{\text{T}}Q_{\text{l}}x_{\text{l}}∕2+q_{\text{l}}^{\text{T}}x_{\text{l}},$$

(16) $$\text{subject to}\;\;\;\;A_{\text{l}}x_{\text{l}}=b_{\text{l}},$$

where $x_{\text{l}}={\dot{\theta }}_{\text{l}}\in R^{n}$, *Q*_1_ = 2*I* ∈ *R^n×n^*, $q_{\text{l}}=\epsilon \left(\theta_{\text{l}}-\theta_{\text{l}}\left(0\right)\right)\in R^{n}$, *A*_l_ = *J*_l_(*θ*) ∈ *R^m×n^*, and $b_{\text{l}}={ṙ}_{\text{l}}$.

Similarly, the bi-criteria optimization subscheme of the right robot manipulator is presented as

(17) $$\text{minimize}\;\;\;\;\;x_{\text{r}}^{\text{T}}Q_{\text{r}}x_{\text{r}}∕2+q_{\text{r}}^{\text{T}}x_{\text{r}},$$

(18) $$\text{subject to}\;\;\;\;A_{\text{r}}x_{\text{r}}=b_{\text{r}},$$

where $x_{\text{r}}={\dot{\theta }}_{\text{r}}\in R^{n}$, *Q*_r_ = 2*I* ∈ *R^n×n^*, $q_{\text{r}}=\epsilon \left(\theta_{\text{r}}-\theta_{\text{r}}\left(0\right)\right)\in R^{n}$, *A*_r_ = *J*_r_(*θ*) ∈ *R^m×n^*, and $b_{\text{r}}={ṙ}_{\text{r}}$.

Finally, the presented two QPs for the left and right robot manipulators of two arms are unified into a new QP formulation, i.e.,

(19) $$\text{minimize}\;\;\;\;\;z^{\text{T}}\mathrm{Wz}∕2+\omega^{\text{T}}z,$$

(20) subject toCz=d,

where coefficient matrices (or vectors) are defined as below:

$$\begin{array}{ll} & z=\left[\begin{array}{c} x_{\text{l}}\\ x_{\text{r}} \end{array}\right]\in R^{2n},\;W=\left[\begin{array}{cc} Q_{\text{l}} & 0\\ 0 & Q_{\text{r}} \end{array}\right]\in R^{2n\times 2n},\\ & \omega =\left[\begin{array}{c} q_{\text{l}}\\ q_{\text{r}} \end{array}\right]\in R^{2n},\;C=\left[\begin{array}{cc} J_{\text{l}}\left(\theta \right) & 0\\ 0 & J_{\text{r}}\left(\theta \right) \end{array}\right]\in R^{2m\times 2n},\\ & \;\;\;\;\;d=\left[\begin{array}{c} b_{\text{l}}\\ b_{\text{r}} \end{array}\right]=\left[\begin{array}{c} {ṙ}_{\text{l}}\\ {ṙ}_{\text{r}} \end{array}\right]\in R^{2m}. \end{array}$$

## 5 Recurrent Neural Network Solver

Note that there are many methods to solve such a standard QP problem. The most common approach is to use a Lagrange multiplier and to minimize a cost function (Li et al., 2013c; Xiao, 2016a). Thus, for dynamic quadratic optimization (equations (19) and (20)), its related Lagrangian is presented as follows:

$$H\left(z,\lambda \right)=z^{\text{T}}\mathrm{Wz}∕2+\omega^{\text{T}}z+\lambda^{\text{T}}\left(\mathrm{Cz}-d\right),$$

where λ ∈ *R*^2^*^m^* denotes the multiplier variable.

It is well known that solving the quadratic optimization (equations (19) and (20)) could be achieved by zeroing the following equations:

$$\left\{\begin{array}{l} \frac{\partial H\left(z,\lambda \right)}{\partial x}=\mathrm{Wz}+\omega +C^{\text{T}}\lambda =0,\;\\ \frac{\partial H\left(z,\lambda \right)}{\partial \lambda \left(t\right)}=\mathrm{Cz}-d=0.\; \end{array}\right.$$

Let

$$\begin{array}{ll} G & =\left[\begin{array}{cc} W & C^{\text{T}}\\ C & 0 \end{array}\right]\in R^{\left(2n+2m\right)\times \left(2n+2m\right)},y=\left[\begin{array}{c} z\\ \lambda \end{array}\right]\in R^{2n+2m},\\ u & =\left[\begin{array}{c} -\omega \\ d \end{array}\right]\in R^{2n+2m}. \end{array}$$

The above linear equations can be further equivalent to the following:

(21) Gy=u.

Note that there were a lot of methods to solve the above linear equation system (equation (21)). In this part, a gradient-based neural network (GNN) is presented and investigated for solving the proposed bi-criteria optimization scheme and the finally equivalent equation (21). By following the literature (Zhang et al., 2009; Xiao and Zhang, 2011; Yi et al., 2011; Li et al., 2013c; Xiao, 2016c), the design procedure of GNN is listed as below.

First, an non-negative scalar-based energy function Ω is defined as follows:

(22) $$\Omega =∥\mathrm{Gy}-u{∥}_{2}^{2}∕2.$$

Second, the negative gradient of Ω can be solved as $-\partial \Omega ∕\partial y=G^{\text{T}}\left(\mathrm{Gy}-u\right)$.

Finally, according to gradient neural network design formula ẏ=−γ∂Ω∕∂y, the GNN model for dynamic inverse kinematics problem can be described as follows:

(23) $$ẏ=-\gamma G^{\text{T}}\left(\mathrm{Gy}-u\right),$$

where *y* ∈ *R*^2^*^n^*^+2^*^m^* denotes the neural state of GNN model (equation (23)).

## 6 Simulative Verifications

**Figure 1:**
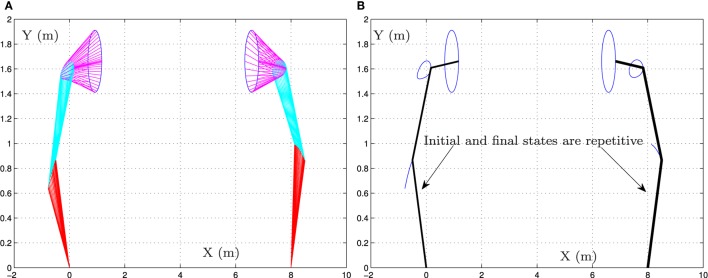
Simulation results when the dual three-link manipulator tracks the given circular path synthesized by the bi-criteria optimization scheme (equations (19) and (20)) and GNN model (equation (23)). **(A)** Motion trajectories of dual manipulator and **(B)** desired circular path and actual trajectory.

**Figure 2:**
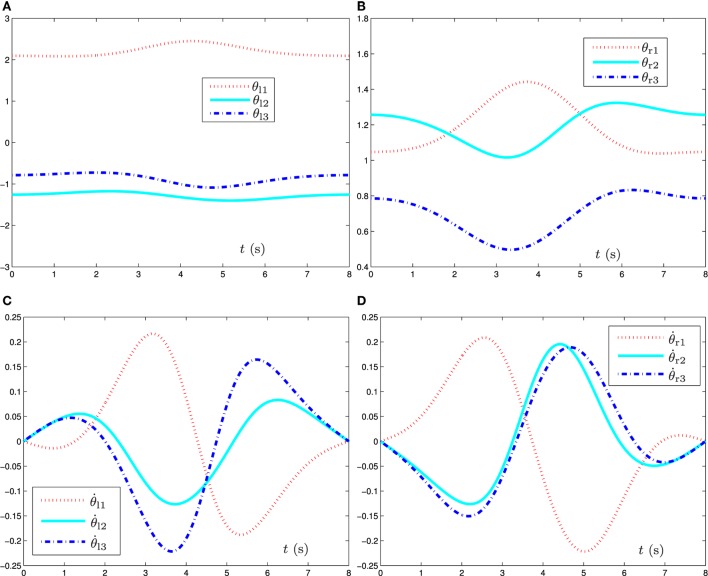
Simulation results when the dual three-link manipulator tracks the given circular path synthesized by the bi-criteria optimization scheme (equations (19) and (20)) and GNN model (equation (23)). **(A)** Joint angle *θ*_l_ profile of left manipulator, **(B)** joint angle *θ*_r_ profile of right manipulator, **(C)** joint velocity ${\dot{\theta }}_{\text{l}}$ profile of left manipulator, and **(D)** joint velocity ${\dot{\theta }}_{\text{r}}$ profile of right manipulator.

**Figure 3:**
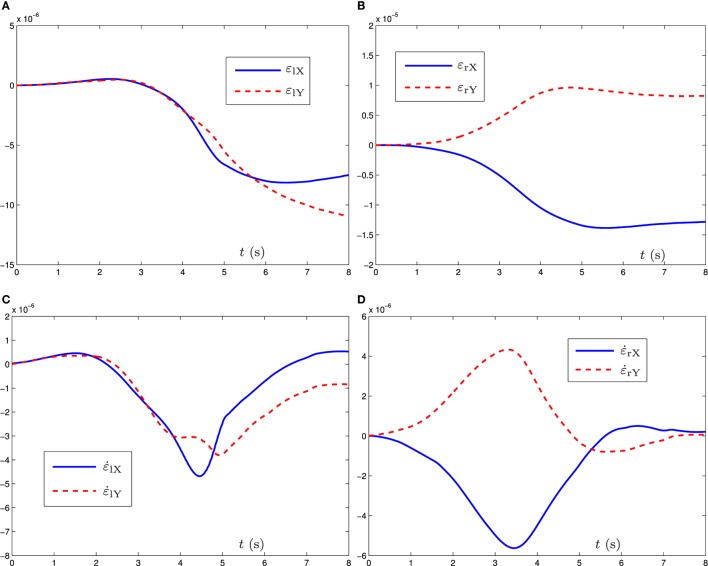
Simulation results when the dual three-link manipulator tracks the given circular path synthesized by the bi-criteria optimization scheme (equations (19) and (20)) and GNN model (equation (23)). **(A)** Position error *ε*_l_ profile of left manipulator, **(B)** position error *ε*_r_ profile of right manipulator, **(C)** velocity error ${\dot{\varepsilon }}_{\text{l}}$ profile of left manipulator, and **(D)** velocity error ${\dot{\varepsilon }}_{\text{r}}$ profile of right manipulator.

In this part, the unified bi-criteria optimization scheme (equations (19) and (20)) is applied to a dual three-link planar manipulator and solved by the presented GNN model (equation (23)). In computer simulations, the end-effectors of the dual manipulators are expected to simultaneously track a circle. Without loss of generality, design parameters ϵ = 10 and γ = 10^7^; the task execution time is 8 s, and the radius of the desired circle is 0.25 m. Besides, the joints of the left and right manipulators are expected to begin with the initial states *θ*_l_(0) = [3π/4, −2π/5, −π/4]^T^ rad and *θ*_r_(0) = [π/3, 2π/5, π/4]^T^ rad, respectively. The computer simulations are illustrated in Figures 1–3, which is solved by the proposed bi-criteria optimization scheme and the presented recurrent neural network.

Specifically, Figure 1 shows the whole motion trajectories of the dual three-link planar manipulators when the end-effectors track the given circular path. As seen from Figure 1A, the circular path-tracking task is performed successfully by the dual three-link planar manipulators. In addition, from Figure 1B, we can see that the final state and the initial state of the dual three-link planar manipulators coincide with each other.

Figure 2 shows the joint-variable (including joint angle and joint velocity) profiles during the task execution of the dual three-link planar manipulators. From this figure, we can conclude that the proposed bi-criteria optimization scheme [synthesized by GNN model (equation (23))] can not only solve the joint-angle drift problem but also prevent the occurrence of high joint velocity in this path-tracking task. Specifically, after the end-effectors completing the circular-path tracking task, the final joint states of the left and right manipulators return to their initial states, which can be seen in Figures 2A,B. In addition, from Figures 2C,D, we can observe that the situation of the high joint velocity does not happen, and the final velocity of each joint for the dual three-link manipulators is equal to zero. It is worth pointing out that, if the final joint velocities is not equal to zero, the manipulator’ joints will not stop immediately at the end of the task duration; and thus, the non-repetitive problem would happen. These results demonstrate and verify the effectiveness of such a bi-criteria optimization scheme synthesized by GNN model (equation (23)).

For further verifying the accuracy of the proposed bi-criteria optimization scheme and GNN model (equation (23)), Figure 3 shows the corresponding position error *ε*(*t*): = *r*(*t*) − *f* (*θ*(*t*)) and the velocity error $\dot{\varepsilon }\left(t\right)$ of the left robot manipulator and the right robot manipulator, where *ε_X_* and *ε_Y_* denote, respectively, the X-axis and Y-axis components of *ε*(*t*). As observed from Figures 3A,B, the corresponding X-axis and Y-axis components of position errors for the left robot manipulator and the right robot manipulator are less than 2 × 10^−5^ m. Besides, from Figures 3C,D, we can obtain that the X-axis and Y-axis components of velocity errors for the left robot manipulator and the right robot manipulator are less than 6 × 10^−6^ m. These demonstrate that the given circular path tracking task is fulfilled well *via* the proposed velocity-level bi-criteria optimization scheme.

In summary, the end-effector tasks are performed very well by synthesizing the proposed velocity-level bi-criteria optimization scheme. The detailed results verifies the effectiveness and applicability of the proposed bi-criteria optimization scheme for coordinated path tracking of dual redundant robot manipulators using the recurrent neural network.

## 7 Conclusion

In this article, a novel velocity-level bi-criteria optimization scheme (i.e., integrating minimum velocity norm and repetitive motion) has been proposed and investigated for complex motion planning of dual robot manipulators. Such a bi-criteria optimization scheme can not only prevent the occurrence of high joint-velocity but also remedy the joint angle drifts of dual redundant robot manipulators well. In addition, the proposed scheme guarantees the joint velocity equals zero at the end of path tracking motion. To do so, two subschemes have been presented for the left and right robot manipulators, which are reformulated as two general quadratic programs (QPs). Then, such two general QP problems have been further unified into one standard QP formulation. Simulative results based on the dual three-link robot manipulators have substantiated the efficacy and applicability of the proposed velocity-level bi-criteria optimization scheme. The future work may lie in the applications of the bi-criteria optimization scheme to real robot manipulators.

## Author Contributions

LX: experiment preparation, data acquisition and processing, and publication writing; YZ: experiment preparation, data processing, and publication drafting; BL: experiment technology support and publication review; ZZ and LD: experiment preparation and publication review; LJ: experiment preparation, data acquisition, and publication review.

## Conflict of Interest Statement

The authors declare that the research was conducted in the absence of any commercial or financial relationships that could be construed as a potential conflict of interest.
